# A family of orthologous proteins from centipede venoms inhibit the hKir6.2 channel

**DOI:** 10.1038/s41598-019-50688-x

**Published:** 2019-10-01

**Authors:** Yajamana Ramu, Zhe Lu

**Affiliations:** Department of Physiology, Perelman School of Medicine, University of Pennsylvania, Philadelphia, PA 19104 USA

**Keywords:** Ion transport, Potassium channels

## Abstract

Inhibitors targeting ion channels are useful tools for studying their functions. Given the selectivity of any inhibitor for a channel is relative, more than one inhibitor of different affinities may be used to help identify the channel in a biological preparation. Here, we describe a family of small proteins in centipede venoms that inhibit the pore (hKir6.2) of a human ATP-sensitive K^+^ channel (hK_ATP_). While the traditional peptide-sequencing service gradually vanishes from academic institutions, we tried to identify the sequences of inhibitory proteins purified from venoms by searching the sequences of the corresponding transcriptomes, a search guided by the key features of a known hKir6.2 inhibitor (SpTx1). The candidate sequences were cross-checked against the masses of purified proteins, and validated by testing the activity of recombinant proteins against hKir6.2. The four identified proteins (SsdTx1-3 and SsTx) inhibit hK_ATP_ channels with a K_d_ of <300 nM, compared to 15 nM for SpTx1. SsTx has previously been discovered to block human voltage-gated KCNQ K^+^ channels with a 2.5 μM K_d_. Given that SsTx inhibits hKir6.2 with >10-fold lower K_d_ than it inhibits hKCNQ, SsTx may not be suitable for probing KCNQ channels in a biological preparation that also contains more-SsTx-sensitive K_ATP_ channels.

## Introduction

ATP-sensitive K^+^ (K_ATP_) channels were discovered more than three decades ago in cardiac myocyte^1^, which were subsequently found in pancreatic β cells that secret insulin^2–5^. The K_ATP_ channels in β cells are believed to couple the blood glucose level to insulin secretion^6–8^. Gain-of-function mutants of Kir6.2 over-expressed in mice or spontaneously in humans leads to *permanent neonatal diabetes mellitus* (PNDM)^9–13^. In the β cells, a subtype of inward-rectifier K^+^ channels, Kir6.2, and a subtype of sulfonylurea receptors, SUR1, in a four-to-four stoichiometry, form a K_ATP_ channel that is inhibited by intracellular ATP^14–19^.

Inhibitors of ion channels have served as important tools to understand the physiology, pathophysiology, and the structure-function relationship of individual members of this important class of biological molecules. Venoms of animals, which contain a large number of small proteins commonly called toxins, prove to be a rich source of inhibitors against ion channels. We previously discovered a 54-residue venom protein, dubbed SpTx1, in the venom of *Scolopendra polymorpha* for its inhibitory activity against hKir6.2^20^, a centipede species that is found to inhabit the Southwestern part of the United States. We were curious to learn whether centipedes from another continent would contain orthologous proteins of SpTx1 that also inhibit hKir6.2.

For the reason to be discussed, we tested here the approach of using the information regarding a short, functionally important region of SpTx1 to guide our identification of the sequences of additional inhibitors, which are present in the venoms of other centipede species whose transcriptome sequences are available. We then verified the identified sequences by mass spectroscopy and examination of the inhibitory activities of the corresponding recombinant proteins.

## Results

### Searching for hKir6.2-inhibiting activity and biochemical purification

The interaction between SUR1 and Kir6.2 is normally necessary for Kir6.2 to reach the cell membrane^21^. However, if the C-terminal 26 residues in the Kir6.2 polypeptide chain are deleted, the resulting mutant Kir6.2 channel (dubbed Kir6.2-ΔC26) alone can reach the membrane without the co-expression of SUR1^22^. This mutant channel has a markedly reduced ATP sensitivity. Also, to a varying degree, many PNDM-causing point mutations lower Kir6.2’s sensitivity to ATP, among which the V59G mutation nearly abolishes the sensitivity^23^. Thus, we combined the two mutations, ΔC26 and V59G, to create a mutant channel that not only expresses as a functional channel on its own but also conducts robust current even in the presence of millimolar concentrations of intracellular ATP in an intact cell. The channel with this background double-mutation ΔC26-V59G, dubbed Kir6.2^bgd^, served as a convenient preparation for us to search for and study the blocker of the Kir6.2 pore here in a heterologous expression system.

**Figure 1:**
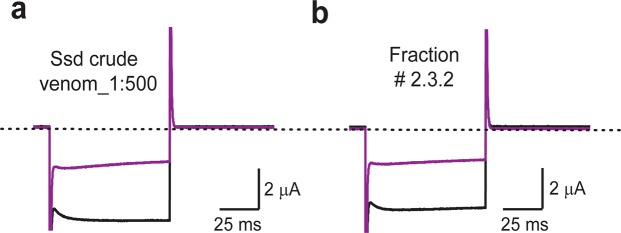
Inhibition of hK_ATP_ channels by *Scolopendra subspinipes dehaani* venom and a purified venom protein. (**a**,**b**) Currents of hK_ATP_ activated by adding 3 mM azide to a 100 mM K^+^ containing bath solution and recorded in the absence (black traces) or presence (violet traces) of 1:500 dilution of crude venom **(a)** or the HPLC fraction indicated by asterisk in Fig. 2d **(b)**. The currents were recorded by stepping voltages from the holding potential 0 mV to −80 mV. The dotted lines indicate zero current levels.

**Figure 2:**
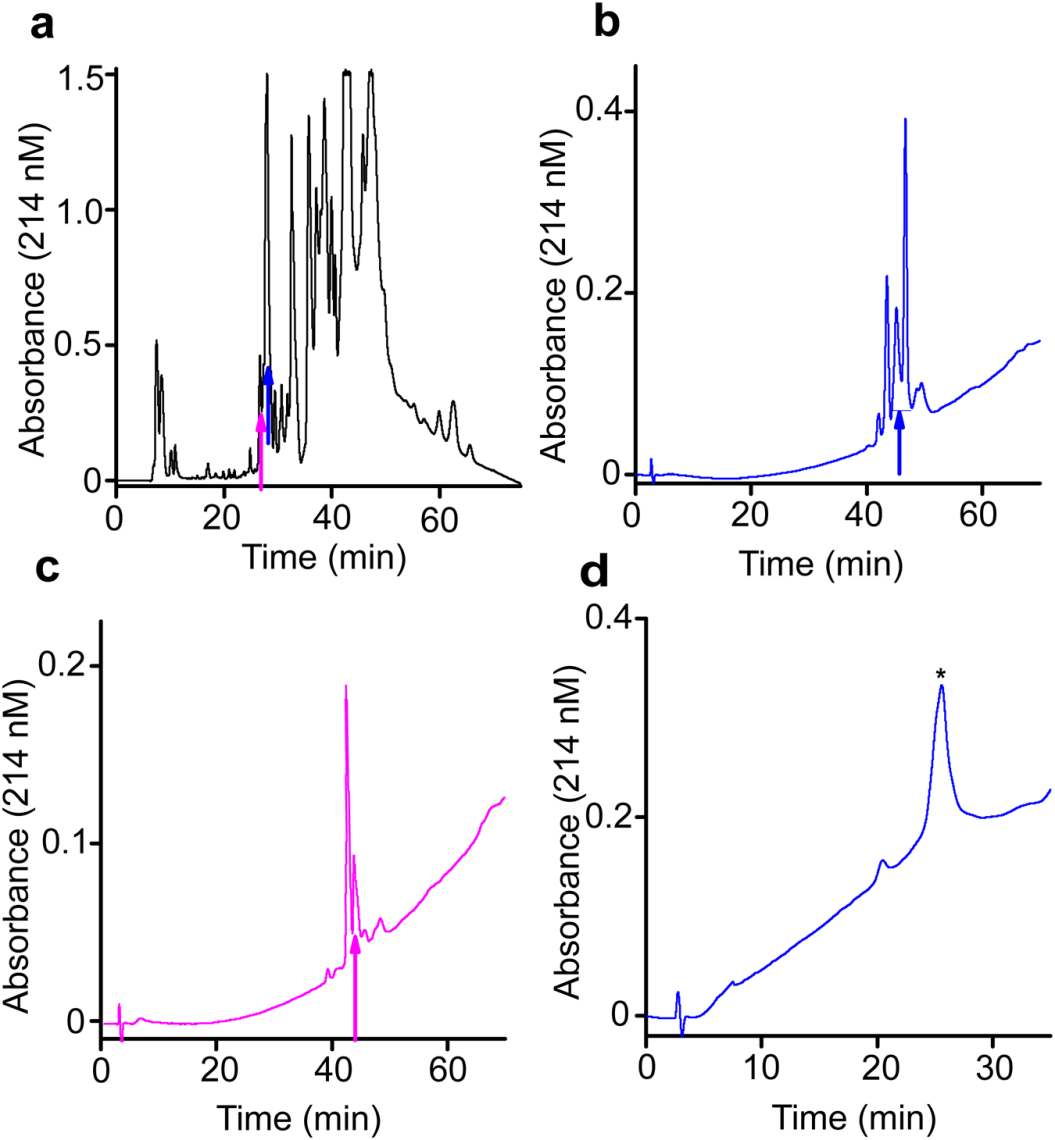
Purification of hK_ATP_-inhibiting materials from *Scolopendra subspinipes dehaani* venom by HPLC. **(a**) Crude venom was fractionated on a semi-preparative C18 column with an acetonitrile gradient. The active peaks are indicated by magenta and blue arrows. (**b**,**c**) The peak indicated by the meganta or blue arrow in (**a**) was purified on an analytical C18 column with a methanol gradient. **(d)** The peak indicated the blue arrow in (**b**) was further purified on the analytic C18 column with an organic mobile phase containing 1:2 mixture of methanol and isopropanol.

Figure 1a shows that the venom of the centipede *Scolopendra subspinipes dehaani* (Ssd; a species that inhabits Asia) suppressed the current through hK_ATP_ channels, formed by hKir6.2 co-expressed with hSUR1, in *Xenopus* oocytes. To biochemically identify the underlying inhibitory proteins, we purified the venom proteins through sequential steps of HPLC chromatography and tested the activities of the resulting fractions (Figs 1b and 2). The crude venom was first fractionated on a semi-preparative reversed-phase C18 column (Fig. 2a). Two of the resulting fractions, indicated by the magenta and blue arrows, contained inhibitory activity against hK_ATP_ channels and were further fractionated on an analytical C18 column (Fig. 2b,c).

In this second round of HPLC purification, the fraction, indicated by the blue arrow in Fig. 2a, was fractioned to three major peaks, among which the middle, smallest peak contained the inhibitory activity, labeled as SsdTx1 for latter reference (Fig. 2b). An adjacent peak also contained weaker inhibitory activity, called SsdTx2. The fraction, which is indicated by the magenta arrow in Fig. 2a, exhibited two major peaks, between which the second, smaller peak contained the inhibitory activity, dubbed SsdTx3 (Fig. 2c). Like SpTx1, the materials that underlie these active peaks have molecular masses ~6 kDa determined on mass spectrometry.

On the basis of our present experience in purifying a minimal amount of these active materials for mass analysis and previous experience in purifying and identifying SpTx1, it would be practically unfeasible for us to determine the full sequence of any of the three potential inhibitors by the Edman degradation method. For SsdTx2 and SsdTx3, we might not even be able to obtain an adequate amount of purified materials to obtain partial sequences.

### Determination of critical basic residues in SpTx1

**Figure 3:**
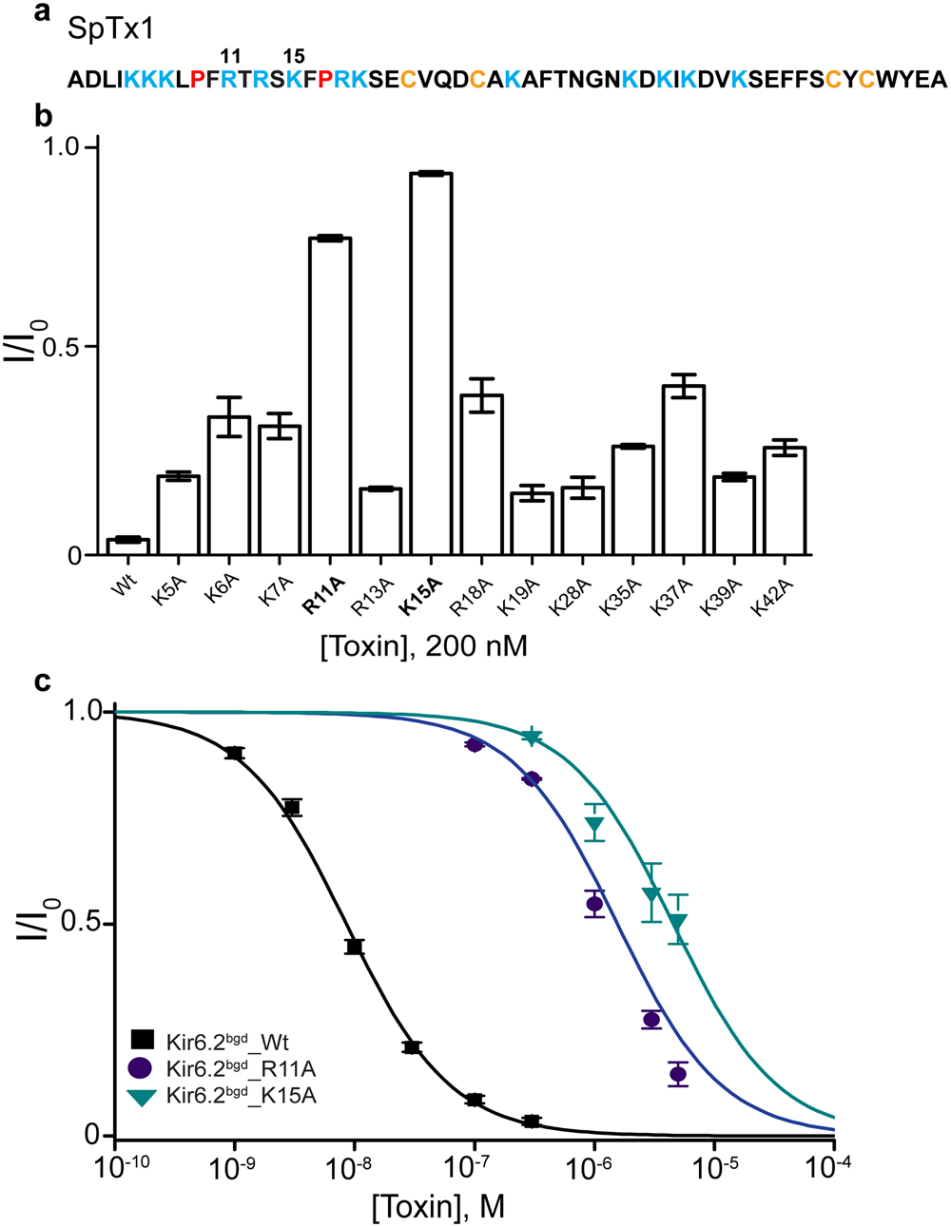
Identification of critical positively charged residues in SpTx1 on its inhibitory potency. **(a)** Primary sequence of SpTx1 where cysteine, proline, and basic residues are colored orange, maroon, and cyan respectively; the positions of two critical residues are indicated by their numbers (11 and 15). (**b**) Fractions of channel currents (I/I_o_) of Kir6.2^bgd^ in the presence of wild-type or mutant SpTx1, each at 200 nM. All data are plotted as mean ± s.e.m. (*n* = 3–6). **(c)** Fractions of channel currents plotted against the concentration of wild-type and mutant SpTx1. The curves superimposed on data correspond to the fits of an equation for a bimolecular reaction, yielding K_d_ values of 8.52 (±0.37) × 10^−9^ M for SpTx1-Wt, 1.6 × (±0.075) 10^−6^ M for SpTx1-R11A, and 4.55 × (±0.55) 10^−6^ M for SpTx1-K15A. All data are plotted as mean ± s.e.m. (*n* = 4–10).

To circumvent the problem, we proceeded to determine the critical residues in SpTx1 (Fig. 3a) underlying its potent inhibition of Kir6.2, so as to use this information to help identify SsdTx1-3 among the several hundreds of transcripts in the venom transcriptome of *Scolopendra subspinipes dehaani*, whose sequences have been determined^24^. As positively charged residues in the venom proteins generally play critical roles in their block of the K^+^ pores^25^, we replaced each of such residues with alanine (Fig. 3a) and tested the resulting mutant SpTx1 against hKir6.2^bgd^. Among the mutations, R11A and K15A had the largest effects on lowering the potency of SpTx1 (Fig. 3b). Analysis of the dose-inhibition curves yielded the K_d_ values of 1.6 μM and 4.5 μM for the channels to interact with SpTx1 carrying the R11A and K15A mutation, compared to a K_d_ value of 8.5 nM for wild-type SpTx1 (Fig. 3c).

Residues R11 and K15 are located in a segment delineated by two proline residues, here dubbed the P-P segment (Fig. 3a). We thus used this positively charged residue-containing segment and the feature of four cysteines to identify SsdTx1-3.

### Identification of inhibitors from the venom transcriptome

**Figure 4:**
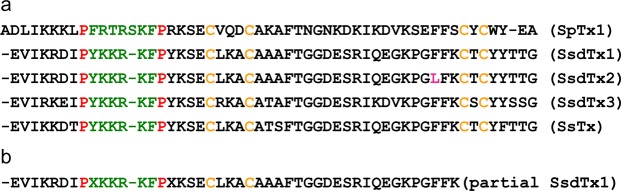
Sequences of venom proteins that inhibit hK_ATP_ channels. **(a)** Sequences of SsdTx1 (KC145039, GenBank), SsdTx2 (KC144386), SsdTx3 (KC144884), and SsTx (PDB 5X0S) aligned with SpTx1. The proline residues in the P-P segment are colored maroon, those residues between them colored green, and cysteine residues colored orange; residue L43 in SsdTx2 is colored magenta. (**b**) Partial amino acid sequence of SsdTx1 determined by Edman sequencing, which corresponds to the peak indicated by asterisk in Fig. 2d. Dashes in (**a**,**b**) were used to create gaps to better align the sequences.

Going through the published transcript sequences of the venom transcriptome of *Scolopendra subspinipes dehaani* one-by-one^24^, we found three transcripts that have both four cysteines and a P-P segment harboring positively charged residues (Fig. 4a; experimentally determined partial sequence of SsdTx1 is shown in Fig. 4b). One of them has the predicted mass of 6031.98 Daltons, which is about four Daltons greater than the experimentally determined value of 6027.74 for SsdTx1 (blue arrow-pointed peak, Fig. 2b). This difference would be consistent with the formation of two disulfide bonds among the four cysteine residues, as is the case of SpTx1^20^. Another transcript has the same sequence as the putative SsdTx1, except for position 43 where it is leucine instead of phenylalanine in SsdTx1. This second transcript most likely corresponds to SsdTx2, the activity associated with the peak preceding that of SsdTx1 (Fig. 2b). The predicted mass of the third transcript (6105.08 Daltons) is about four Daltons greater than the observed mass (6101.15 Daltons) of SsdTx-3 (magenta arrow-pointed peak, Fig. 2c), again a difference that is consistent with the formation of two pairs of disulfide bonds.

### Inhibitory activity of recombinant SsdTx 1-3 proteins against hKir6.2

**Figure 5:**
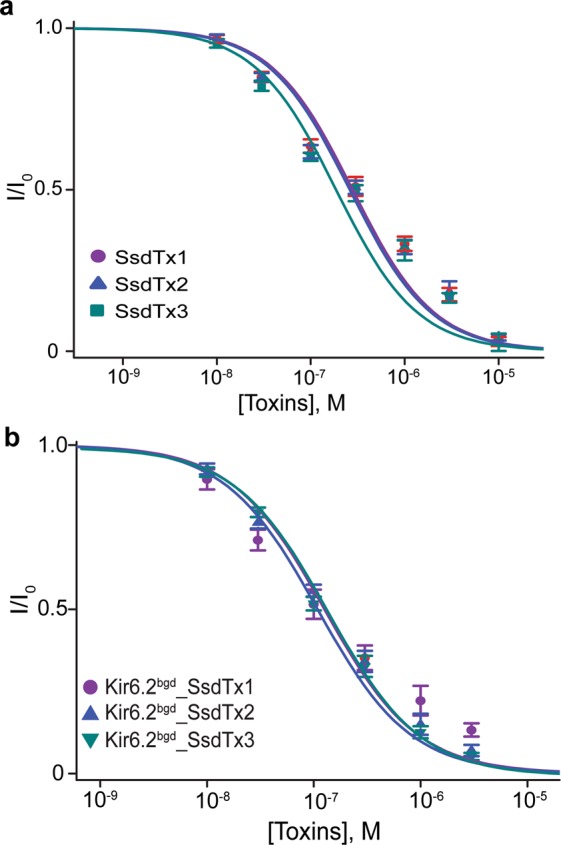
Inhibition of hK_ATP_ and hKir6.2^bgd^ channels by SsdTx1, SsdTx2 and SsdTx3. **(a)** Fractions of hK_ATP_ currents plotted against the concentration of SsdTx1, SsdTx2, or SsdTx3. The curves superimposed on data correspond to the fits of an equation for a bimolecular reaction, yielding K_d_ values of 2.78 × (±0.62) 10^−7^ M for SsdTx1, 2.60 (±0.52) × 10^−7^ M for SpTx2, and 2.83 × (±0.46) 10^−7^ M for SsdTx3. **(b)** Fractions of hKir6.2^bgd^ currents plotted against the concentration of SsdTx1, SsdTx2, or SsdTx3. The curves superimposed on data correspond to the fits of an equation for a bimolecular reaction, yielding K_d_ values of 1.26 × (±0.35) 10^−7^ M for SsdTx1, 1.23 (±0.10) × 10^−7^ M for SpTx2, and 1.18 × (±0.07) 10^−7^ M for SsdTx3. All data are plotted as mean ± s.e.m. (*n* = 5–7).

To determine whether SsdTx1-3 were indeed the inhibitors against hK_ATP_ channels, we produced the recombinant proteins based on the identified sequences. An analysis of the dose-inhibition curves against hK_ATP_ channels revealed a K_d_ of 278 nM, 260 nM, or 281 nM for SsdTx1, SsdTx2 or SsdTx3 (Fig. 5a). We have thus identified SsdTx1-3 as inhibitors against hK_ATP_ channels, with K_d_ values differing from SpTx1. Furthermore, we tested the three inhibitors against the current of Kir6.2^bgd^ (without co-expression with hSUR1). All of them caused comparable levels of inhibition and had similar K_d_ values (Fig. 5b). Thus, like SpTx1, these inhibitors also target hK_ATP_ channels by blocking hKir6.2.

Following the positive identification of the inhibitors against hKir6.2, we went back to biochemistry, solely for additional confirmation of the validity of the present approach. We further purified SsdTx1 through a third round of HPLC and were able to accumulate a sufficient amount of the active material for performing the Edman sequencing, through total 28 runs of repeated, sequential HPLC purification of samples (Figs 1b, 2d). This analysis revealed the sequence of N-terminal 45 residues of SsdTx1 (Fig. 4b), which indeed corresponds to that of the transcript identified as SsdTx1 (Fig. 4a).

### hKir6.2-inhibiting activity of SsTx previously known to inhibit hKCNQ channels

**Figure 6:**
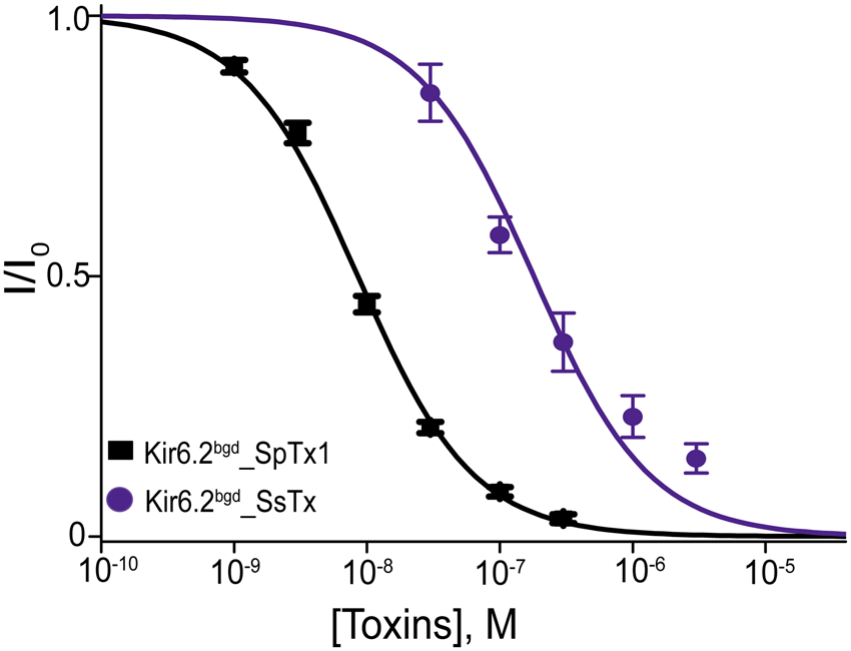
Comparison of inhibition of hKir6.2^bgd^ by SpTx1 and SsTx. Fractions of channel currents plotted against the concentration of SpTx1 or SsTx. The curves superimposed on data correspond to the fits of an equation for a bimolecular reaction, yielding K_d_ values of 8.52 (±0.37) × 10^−9^ M for SpTx1, and 1.80 × ( ± 0.42) 10^−7^ M for SsTx. All data are plotted as mean ± s.e.m. (*n* = 5).

Using the sequences of those Kir6.2 inhibitors as baits, we fished out SsTx from the sequence deposited in the gene bank. SsTx is a 53-residue protein naturally present in the venom of *Scolopendra subspinipes mutilans*, and has previously been identified as an inhibitor against various hKCNQ isoforms, with practically the same micromolar affinity (K_d_ of ~2.5 μM)^26^. SsTx has four cysteine residues and a P-P segment that contains several positively charged residues (Fig. 4a). These features predict that SsTx would inhibit the current through the hKir6.2 pore. An analysis of the dose-inhibition relation yields a K_d_ value of 180 nM for SsTx, compared to 8.5 nM for SpTx1 (Fig. 6). Despite the fact that it blocked the Kir6.2 pore with lower affinity than SpTx1 did, SsTx still blocked the Kir6.2 pore with much higher affinity than it blocked the hKCNQ pore.

**Figure 7:**
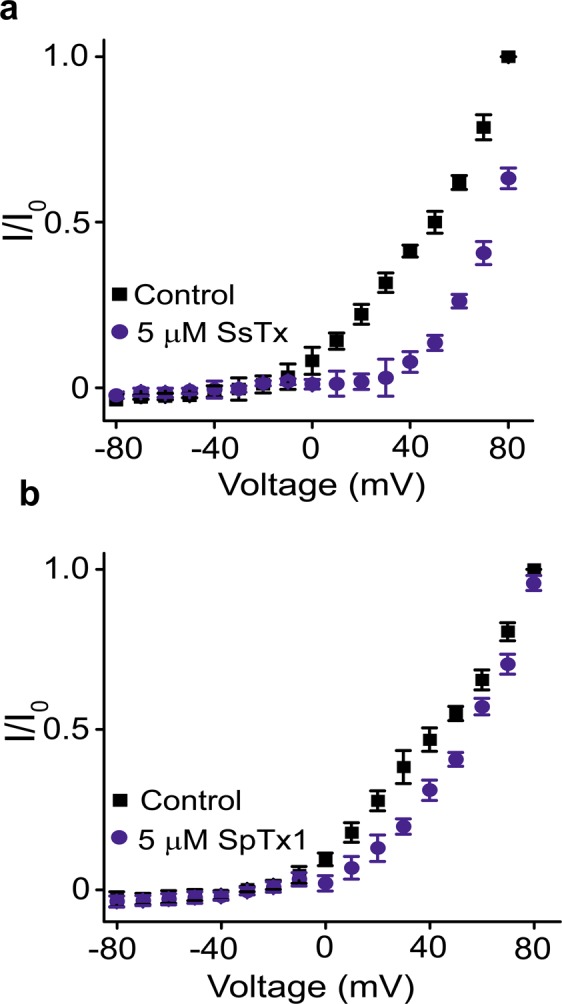
Comparison of inhibition of hKCNQ1 channel currents by SpTx1 and SsTx. (**a**,**b**) I-V curves of hKCNQ1 co-expressed with hKCNE1 obtained in the absence (control) or presence of 5 μM SsTx **(a)** or 5 μM SpTx1 **(b)**. All data are plotted as mean ± s.e.m. (*n* = 6–8). The bath solution contained 5 mM KCl and 95 mM NaCl.

We also tested SpTx1 against the hKCNQ1 channels, co-expressed with hKCNE1, in *Xenopus* oocytes. Less inhibition by SpTx1 than SsTx occurred at all positive voltages where meaningful currents were elicited (Fig. 7). In any case, both inhibitors blocked the pore of hKir6.2 much more potently than they blocked hKCNQ1 pore.

## Discussion

In the present study, we have shown that the venoms of multiple centipede species contain one or more proteins that target hK_ATP_ channel by blocking the hKir6.2 pore. This family of five proteins, thus far identified, has the conserved features of four cysteine residues and a P-P segment that contains positively charged residues important for blocking the K^+^ pore (Fig. 4). As always, the selectivity of inhibitors among different types of K^+^ channel is relative. SsTx has previously been shown to block the KCNQ pore with a K_d_ of 2.5 μM. We have now found that SpTx1 blocks the pore with a slightly lower affinity, but both of them block the hKir6.2 pore with much higher affinities (a K_d_ of 15 nM for SpTx1 or 180 nM for SsTx).

Judging from the relevent K_d_ values, SsTx would profoundly block the hKir6.2 channel at a dose that begins to produce any meaningful inhibitory effect on hKCNQ channels. Thus, caution must be exercised when using SsTx as a tool to probe hKCNQ channels in a preparation that contains SsTx-sensitive hKir6.2 channels. However, SpTx1 is not expected to have any meaningful impact on hKCNQ channels at a dose that would cause marked inhibition of hKir6.2 channels. Furthermore, one can use the high-affinity SpTx1 in conjunction with another inhibitor of lower affinity (SsdTx1-3), instead of SpTx1 alone, to identify hKir6.2 in a biological preparation, on the basis of the expected different K_d_ values of the two inhibitors.

The difficulty in discovering a new toxin targeting a given channel has been twofold. First, the probability to find a venom that contains the inhibitory activity is often very low. Second, it can be a very challenging process to biochemically identify the inhibitor. Such identification requires all individual inhibitory proteins be purified to near homogeneity and then their sequences be determined typically by the Edman degradation methods^27^, which may not be practically achievable, especially for those containing more than 50 residues. Unfortunately, the Edman degradation method is abandoned by more and more academic service facilities. Over the years, we have migrated from Harvard Medical School’s facility to Yale University’s, and then to Stanford University’s for sequencing service, all of which have sequentially ceased their Edman degradation sequencing services.

As we are losing high-quality sequencing services at academic institutions (staffed with expert consulting scientists), we had to become more resourceful in the process of identifying toxins targeting ion channels. Here, we tested the feasibility of using the information learned from a known inhibitor to guide our discovery of new inhibitors by searching the sequences of a transcriptome, and cross-checked the identified candidates using experimental molecular masses of purified active venom components. Determination of the mass of a protein generally requires a test sample merely in pmole quantity, which does not need to be highly pure because the masses of individual proteins in a mixture can usually be determined by mass spectrometry. Finally, we confirmed the positive identification of useful inhibitors by producing active recombinant proteins. The present study illustrates a successful example of identifying inhibitors in a venom against a chosen channel without relying on protein peptide sequence information.

## Methods

### Mutagenesis and electrophysiological recordings

The hKir6.2 and hSUR1 cDNAs were subcloned in pGEMHE, whereas hKCNQ1 and hKCNE1 were in the pSP64 vector. All mutant cDNAs were produced through PCR-based mutagenesis and confirmed by DNA sequencing. cRNAs were synthesized with T7 polymerase or SP-6 polymerase using the corresponding linearized cDNAs as templates. A two-electrode voltage clamp amplifier (Oocyte Clamp OC-725C, Warner Instruments Corp.) was used to record channel currents from *Xenopus* oocytes injected with the cRNA encoding a specified wild-type or mutant channel. To activate K_ATP_ channel currents, 3 mM azide was added to the bath solution. The resistance of electrodes filled with 3 M KCl was 0.2–0.4 MΩ. To elicit current through wild-type or mutant hKir6.2 pores, the membrane potential of oocytes was stepped from the holding potential of 0 mV to −80 mV then to 0 or 80 mV. The bath solution contained (in mM): 100 KCl, 0.3 CaCl_2_, 1.0 MgCl_2_, and 10 HEPES, and BSA (50 μg/mL), where pH was titrated to 7.6. To elicit current through hKCNQ1 co-expressed with hKCNE1, the membrane potential was stepped from the holding potential of −80 mV to 80 mV in 10 mV increments and then to −30 mV. The bath solution contained (in mM): 95 KCl, 5 NaCl, 0.3 CaCl_2_, 1.0 MgCl_2_, 10 HEPES, and BSA (50 μg/mL), where pH was titrated to 7.6. All recombinant inhibitors were produced as previously described for SpTx1^20^. We used a gravity flow system in inhibitor titration studies.

### Biochemical purification of inhibitors from the *Scolopendra subspinipes dehaani* venom

In each of 28 runs, 40 uL of *Scolopendra subspinipes dehaani* crude venom (Spider Pharm) was loaded onto a Semi-preparative (Beckman) reverse-phase C18 column and the fractions were eluted with a linear water-to-acetonitrile gradient at a changing rate of 1% per minute; the flow rate of the mobile phase was 1 mL per minute. All the peaks were collected, dried and re-suspended before assaying them for activity. Active fractions were further purified in two sequential HPLC steps with an analytical C18 column, where samples were eluted with a linear water-to-methanol gradient, or a gradient of water to 1:2 mixture of methanol and isopropanol, each of which had a changing rate of 1% per minute; the flow rate of the mobile phase was 1 mL per minute. The masses of purified materials were determined on MALDI-TOF (Wistar Institute, Proteomics facility). The material purified as shown in Fig. 2d was sequenced by Edman degradation (Stanford University, Protein and Nucleic acid (PAN) facility), which yielded the sequence of N-terminal 45 residues (Fig. 4b).

## Data Availability

The datasets used in the present study are available from the corresponding author on reasonable request.
